# Factors associated with post-traumatic stress disorder among SIMAD University students in Mogadishu, Somalia: a cross-sectional study

**DOI:** 10.1186/s12889-025-24803-9

**Published:** 2025-10-22

**Authors:** Jaweriya Bashir Ahmed, George Nyadimo Agot

**Affiliations:** 2 Faculty of Medicine and Health Science, SIMAD University, Mogadishu, Somalia; 1 Department of Public and Global Health, Faculty of Health Sciences, University of Nairobi, Nairobi, Kenya

**Keywords:** PTSD, Trauma exposure, University students, Somalia, Mental health, Social support

## Abstract

**Background:**

Post-traumatic stress disorder (PTSD) is a psychiatric condition that may develop following exposure to severe or life-threatening traumatic events. Despite ongoing insecurity in Somalia, data on PTSD among university students are limited. This study assessed the occurrence of probable PTSD and its associated factors among students at SIMAD University in Mogadishu.

**Methods:**

An institutional-based cross-sectional study was conducted among 343 students selected through stratified random sampling. PTSD symptoms were assessed using the PTSD Checklist for DSM-5 (PCL-5), applying a cut-off score of 33 to identify probable PTSD. Binary logistic regression analysis was used to identify factors associated with probable PTSD, with statistical significance set at *p* < 0.05.

**Results:**

A total of 343 students participated (mean age 20.2 years; 48.1% female). Overall 34.4% of participants met the criteria for probable PTSD. Female students had significantly higher odds of probable PTSD (aOR = 5.36; 95% CI: 2.77–10.13; *p* < 0.001). Experiencing the death of a friend from a gunshot (aOR = 2.22; 95% CI: 1.13–4.38; *p* = 0.021) and being involved in a transportation accident (aOR = 2.30; 95% CI: 1.05–5.03; *p* = 0.037) were also independently associated with probable PTSD. No significant association was observed with social support.

**Conclusion:**

Probable PTSD affected over one-third of surveyed students, underscoring the psychological burden of ongoing insecurity in Mogadishu. Female gender, interpersonal loss from gun violence, and transportation-related trauma emerged as key associated factors. These findings highlight the urgent need for trauma-informed, gender-sensitive mental health interventions in Somali university settings.

## Introduction

Post-traumatic stress disorder (PTSD) is a mental health condition that develops following direct or indirect exposure to traumatic or stressful events. According to the Diagnostic and Statistical Manual of Mental Disorders (5th ed), it is characterized by symptoms including flashbacks, avoidance, negative thoughts, and heightened reactivity that persist for more than one month, though symptoms may also emerge after a delay [1].

Globally, the lifetime prevalence of PTSD is estimated at 3.9%, increasing to 5.6% among trauma-exposed individuals [2]. However, this burden varies widely depending on context. In regions affected by armed conflict, the prevalence is markedly higher. For instance, a systematic review reported PTSD rates of 30% in conflict-exposed areas of sub-Saharan Africa compared to 8% in non-conflict regions [3]. Similarly, studies in post-conflict settings found a 33.3% prevalence in Southern Lebanon [4] and 23.8% among war survivors worldwide [5]. These findings underscore the profound psychological impact of conflict and displacement.

Several factors have been identified as increasing the likelihood of PTSD following trauma. These include being female [6], of a younger age [7], having a lower socioeconomic status [8], lacking social support [9, 10], and having a history of childhood adversity [11]. In addition, both the number and type of traumatic events experienced are significant determinants of PTSD risk [5]. Social support is also considered a key moderating factor, although cultural norms, individual resilience, and the severity of trauma shape its protective effects [12, 13].

Within this broader context, Somalia provides a particularly relevant setting for PTSD research. The country has endured prolonged armed conflict, political instability, recurrent droughts, and humanitarian crises for over three decades. In 2022 alone, more than 6,500 deaths were linked to conflict-related violence [14]. While several studies have assessed mental health outcomes among Somali refugees [15, 16] there remains limited research on PTSD among individuals who continue to live in Somalia, especially youth. Somali adolescents and young adults face chronic exposure to insecurity, coupled with limited access to mental health services and weakened social structures[17].

University students are an important and accessible subgroup within this population. In conflict-affected regions, students face additional psychological stress due to academic pressure, restricted opportunities, and continued exposure to violence. Previous studies conducted among university students in Syria and Iraq found PTSD prevalence rates of 28.2% and 22.9%, respectively [18, 19]. These studies also identified trauma history, lack of social support, and interpersonal loss as significant predictors of PTSD [20]. However, evidence from Somalia is lacking, despite its prolonged instability.

To address this gap, the present study aimed to examine the burden and correlates of probable PTSD among university students in Mogadishu, Somalia. Specifically, it investigated the relationship between PTSD and sociodemographic characteristics, trauma exposure, and levels of perceived social support. The findings are intended to inform context-specific mental health interventions and contribute to evidence-based policy responses targeting conflict-affected youth in Somalia.

## Methodology

### Study design

This study employed an institutional-based analytical cross-sectional design to investigate factors associated with posttraumatic stress disorder (PTSD) among students attending SIMAD University in Mogadishu, Somalia.

### Study area and population

The study was conducted at SIMAD University, a higher education institution located in Mogadishu, Somalia. Established in 1991, the university offers a range of undergraduate and graduate degree programs, with English as the primary language of instruction. As of the study period (2023), the total number of students enrolled across SIMAD University’s Mogadishu campus was 3,633. Mogadishu, the capital city of Somalia, is divided into eighteen administrative districts and has experienced protracted conflict and instability since the collapse of the Siad Barre regime in 1991.

In recent years, the city has remained a focal point of insecurity, with recurrent violent incidents attributed to the militant group Al-Shabab. According to ACLED in 2023 alone, Mogadishu experienced several high-profile attacks, including suicide bombings at military academies, armed sieges at public venues such as the Pearl Beach Hotel, and targeted explosions near tea shops and government offices [14]. These events resulted in significant civilian casualties and fostered an atmosphere of chronic fear and trauma, making Mogadishu a pertinent setting for this research.

The target population consisted of male and female university students born and raised in Mogadishu. Eligible participants included those who provided informed consent and met the inclusion criteria. Students previously diagnosed with acute stress disorder (ASD) or depression, those with chronic illnesses, and those not born and raised in Mogadishu were excluded to ensure a uniform exposure profile.

### Sample size and sampling strategy

The sample size was calculated using the formula for cross-sectional studies provided by Charan and Biswas [21]:

$$\:n=\:\frac{{Z}_{\alpha\:/2}^{2}*p\:(1-p)}{{d}^{2}}\:\:\:=\:\frac{{\left(1.96\right)}^{2}*0.32*0.68}{{0.05}^{2}}\:=334.24$$

where Z = 1.96 (95% confidence level), *p* = 0.32 (proportion from a prior study [22]), and d = 0.05 (margin of error). This calculation resulted in a sample size of 334, which was adjusted to 352 to account for a 5% non-response rate.

**Fig. 1:**
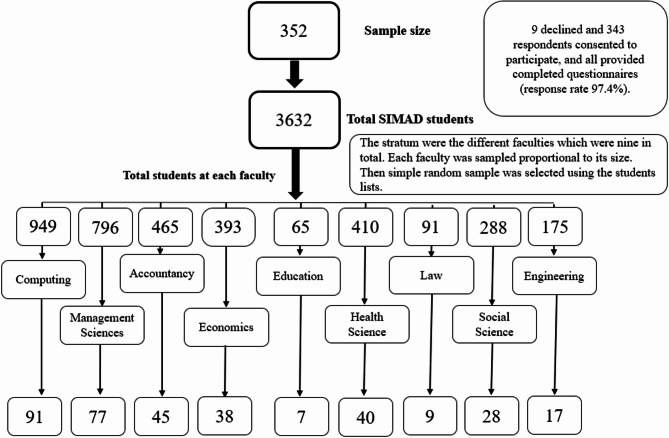
Flow chart of the stratified random sampling

A stratified random sampling approach (Fig. 1) was used to ensure representation across the university’s faculties. Strata were defined by the different faculties, and proportional allocation was applied to determine the number of participants from each stratum. Simple random sampling within each faculty was conducted using the list of enrolled students. Each student was assigned a number, and a random number generator was used to select participants within each faculty.

### Data collection and instruments

Data were collected during November-December 2023 using a structured, self-administered paper-based questionnaire. The process took place in classroom settings during morning hours, under the supervision of the researcher. After a brief explanation of the study and its objectives, students who voluntarily consented were provided with the questionnaire to complete privately at their desks. Participants could choose to complete the survey in either Somali or English. The Somali version was translated by professional translators with expertise in public health and mental health terminology, ensuring linguistic accuracy and cultural relevance.

PTSD status was assessed using the PTSD Checklist for DSM-5 (PCL-5), a validated 20-item screening instrument that measures PTSD symptoms based on DSM-5 diagnostic criteria. Each item was rated on a 5-point Likert scale (0–4), with total scores ranging from 0 to 80. A cutoff score of 33 was used to identify probable cases of PTSD, as supported by prior studies conducted in conflict-affected populations [23]. Other variables included traumatic events, social support and sociodemographic characteristics such as gender, age, marital status, area of residence, and family economic status.

Family economic status was measured using a single-item question with four response options, each reflecting how well a family’s income meets their basic and additional needs. The responses were categorized as follows: (a) poor – income does not cover essential needs; (b) lower-middle – income covers only essential needs; (c) upper-middle – income covers essential needs and some luxury items; and (d) rich – income covers essential needs and all luxury wants. During data collection, essential needs were explained as things like daily meals, rent, school fees, transportation to university, and basic health care. Luxury spending referred to owning the latest smartphone, tablet, or laptop; dining at cafés; purchasing clothes for social occasions; frequent mobile data or airtime use; and hiring a bajaj (three-wheeled motorized taxi) or taxi instead of using a public minibus.

Traumatic events were assessed using a 22-item checklist adapted from the War and Adversity Checklist [24] tailored to capture trauma exposures relevant to the Somali context. The items reflected experiences related to armed conflict (e.g., exposure to gunfire or explosions), interpersonal and community violence (e.g., assaults, robbery), displacement and destruction (e.g., fleeing residence, home demolition), bereavement (e.g., loss of family or friends due to violence or accidents), and transportation-related trauma (e.g., serious accidents or witnessing fatal crashes). Each item was analyzed as a binary variable (Yes/No) to determine whether the participant had experienced that specific event. While the checklist was not locally validated, it was reviewed for contextual relevance.

Social support was measured using the Social Provisions Scale (SPS-10), a validated 10-item scale that evaluates perceived social support [25]. Responses were rated on a 4-point Likert scale (1 = strongly disagree to 4 = strongly agree), with scores categorized as poor (10-20), moderate (21-30), and strong (31-40) social support. Internal reliability was evaluated using Cronbach’s alpha, estimated at α = 0.82 for the traumatic event checklist, α = 0.87 for the PCL-5, and α = 0.80 for the SPS-10, indicating good internal consistency across all measures.

### Data processing and analysis

After collection, the questionnaires were checked on-site for completeness and consistency under close supervision, then entered into an Excel spreadsheet for cleaning. Cleaned and verified data were subsequently exported to STATA version 18.0 for statistical analysis. Descriptive statistics were used to summarize the data: means and standard deviations for continuous variables, and frequencies and percentages for categorical variables.

To identify factors associated with probable PTSD, binary logistic regression analyses were conducted. Univariable analysis was first performed for each independent variable using a liberal p-value threshold of < 0.2 to avoid excluding potential confounders. Variables meeting this criterion were included in the multivariable logistic regression model. However, marital status was excluded from both univariable and multivariable regression analyses due to insufficient variability in the sample, as only four participants were married and none met the criteria for PTSD. Adjusted odds ratios (aOR) with 95% confidence intervals were reported, and statistical significance was set at *p* < 0.05.

## Results

### Participants characteristics

Table 1 presents the sociodemographic characteristics of the participants. A total of 343 students participated in the study. The mean age was 20.15 years (± 2.4), with a median of 20 years and a range of 16–43 years. Most participants (58.9%) were aged 19–21 years. The majority were single (98.8%). Males comprised 51.9% and females 48.1% of the sample. Regarding family economic status, 37.6% reported being in the lower-middle category. In terms of perceived social support levels, 59.77% reported strong support, 36.15% moderate, and only 4.1% reported poor support.

**Table 1:** Sociodemographic and social support characteristics of the participants (n = 343)

Variable	Frequency (*N* = 343)	Percentage (%)
**Age**		
16–18	71	20.7
19–21	202	58.9
22–24	61	17.8
> 24	9	2.6
Mean age: 20.15 Standard Deviation: ±2.4 Median age: 20 Range: 16–43		
**Gender**		
Male	178	51.9
Female	165	48.1
**Marital Status**		
Single	339	98.8
Married	4	1.2
**Family economic Status**		
Poor	21	6.1
Lower middle	129	37.6
Upper middle	116	33.8
Rich	77	22.4
**Area of Residence (Top 10)**		
Yaqshid	81	23.6
Hodan	49	14.3
Warta Nabada	42	12.2
Deyniile	41	12.0
Wadajir	35	10.2
Howl-wadaag	20	5.8
Dharkenley	15	4.4
Karaan	14	4.1
Shibis	12	3.5
Heliwa	11	3.2
**Social Support**		
Poor	14	4.08
Moderate	124	36.15
Strong	205	59.77

### Proportion of probable Post-traumatic Stress Disorder 

Out of 343 respondents, 118 (34.4%) met the criteria for probable PTSD based on the PCL-5 cutoff. The proportion of probable PTSD was higher among females, with 76 out of 165 (46.1%), compared to males with 42 out of 178 (23.6%)

### Frequency of traumatic experiences

The mean number of traumatic experiences was 9.11 (± 3.02). The most frequently reported trauma was being close to explosions or gunfire (80.76%). Other common events included witnessing others injured by bullets (74.34%) and fleeing the area of residence due to insecurity (73.18%). Table 2 provides the full distribution of traumatic events reported.

**Table 2:** Frequency of exposure to traumatic events

No.	Trauma Event	Frequency (*N* = 343)	Percentage (%)
1	Close to Explosion or Gunfire	277	80.76
2	Seen Others Injured by Bullet	255	74.34
3	Fled from Area of Residence	251	73.18
4	Friends/Family Injured by Bullet	194	56.56
5	Exposed to Armed Conflict	191	55.85
6	Family Died from Gunshot or Explosion	174	50.73
7	Threatened with Harm by Armed Forces	160	46.65
8	Witnessed Physical Assault	156	45.48
9	Witnessed Severe Transportation Accident	147	42.86
10	Seen Execution	145	42.27
11	Seen Dead Bodies or Body Parts	143	41.69
12	Been Robbed	141	41.11
13	Home Invasion While Inside	141	41.23
14	Non-Family Physical Assault	136	39.65
15	Experienced Transportation Accident	132	38.60
16	Experienced Explosion	126	36.73
17	Friends Died from Gunshot or Explosion	102	29.74
18	Witnessed Destruction of Residence	65	18.95
19	Family/Friend Died from Transportation Accident	64	18.66
20	Family Physical Assault	61	17.78
21	Home Destroyed Due to Violence	55	16.03
22	Injured by Bullet or Explosion	7	2.04

### Univariable and multivariable binary logistic analysis

Univariable binary logistic regression identified several variables associated with probable PTSD at a liberal threshold of *p* < 0.2. Among sociodemographic factors, only gender met this criterion. Trauma-related variables that were significantly associated with probable PTSD included: experienced explosion, close exposure to gunfire, friends injured by bullet, friends died by gunshot, seen dead bodies, seen execution, home destruction due to violence, fled residence due to violence, threatened by armed personnel, experienced transportation accident, witnessed severe transportation accident, family died in a transport accident, non-family physical assault, family physical assault, been robbed, and home invasion.

Social support was examined but did not show a statistically significant association with probable PTSD in the univariable or multivariable analyses.

Table 3 presents the cross-tabulations of probable PTSD status with each explanatory variable, along with the crude and adjusted odds ratios and their 95% confidence intervals. In the multivariable binary logistic regression, three factors remained statistically significant. Female participants had higher odds of PTSD (aOR = 5.36; 95% CI: 2.77–10.37; *p* < 0.001). Reporting the death of a friend due to gunshot was also associated with increased odds (aOR = 2.22; 95% CI: 1.13–4.38; *p* = 0.021), as was experiencing a transportation accident (aOR = 2.30; 95% CI: 1.05–5.03; *p* = 0.037).

**Table 3:** Cross-tabulations, crude and adjusted odds ratios of factors associated with probable PTSD among Simad University students in Mogadishu (*n* *= 343*)

Variable	PTSD Yes *n* (%)	PTSD No *n* (%)	cOR (95% CI)	aOR (95% CI)	*p*-value
**Age**					
16–18 (ref)	23 (32.4)	48 (67.6)	Ref	Ref	—
19–21	73 (36.1)	129 (63.9)	1.18 (0.67–2.10)	1.16 (0.56–2.38)	0.695
22–24	19 (31.2)	42 (68.8)	0.94 (0.45–1.97)	1.00 (0.39–2.58)	0.995
25–43	3 (33.3)	6 (66.7)	1.04 (0.24–4.55)	2.59 (0.44–15.37)	0.296
**Gender**					
Male (ref)	42 (23.6)	136 (76.4)	Ref	Ref	—
Female	76 (46.1)	89 (53.9)	2.77 (1.74–4.39)	5.36 (2.77–10.37)	< 0.001*
**Area of residence**					
Yaqshiid	23 (30.9)	56 (69.1)	Ref	Ref	
Deyniile	17 (41.5)	24 (58.5)	1.59 (0.73–3.46)	1.92 (0.77–4.80)	0.160
Dharkenley	3 (20.0)	12 (80.0)	0.56 (0.15–2.16)	0.61 (0.13–2.85)	0.527
Hamar-jijab	2 (40.0)	3 (60.0)	1.49 (0.23–9.50)	0.70 (0.08–5.99)	0.741
Hamar-weyne	2 (50.0)	2 (50.0)	2.24 (0.30–16.82)	0.59 (0.04–8.08)	0.690
Heliwa	3 (27.3)	8 (72.7)	0.84 (0.21–3.43)	0.42 (0.09–2.10)	0.293
Hodan	15 (30.6)	34 (69.4)	0.99 (0.46–2.13)	0.81 (0.33–1.98)	0.649
Howl-wadaag	6 (30.0)	14 (70.0)	0.96 (0.33–2.79)	0.73 (0.21–2.50)	0.611
Karaan	3 (21.4)	11 (78.6)	0.61 (0.16–2.38)	0.65 (0.14–3.05)	0.587
Shibis	4 (33.3)	8 (66.7)	1.12 (0.31–4.07)	1.61 (0.37–7.04)	0.526
Waberi	4 (40.0)	6 (60.0)	1.49 (0.39–5.76)	0.43 (0.09–2.09)	0.295
Wadajir	14 (40.0)	21 (60.0)	1.49 (0.65–3.41)	1.00 (0.38–2.64)	0.992
Warta Nabada	18 (42.9)	24 (57.1)	1.68 (0.78–3.64)	1.42 (0.57–3.56)	0.450
**Traumatic events**					
Experienced Explosion					
No(ref)	64 (29.5)	153 (70.5)	Ref	Ref	—
Yes	54 (42.9)	72 (57.1)	1.79 (1.13–2.83)	1.14 (0.60–2.15)	0.690
Close exposure to gunfire					
No(ref)	11 (16.7)	55 (83.3)	Ref	Ref	—
Yes	107 (38.6)	170 (61.4)	3.15 (1.58–6.28)	2.19 (0.97–4.95)	0.060
Friends injured by bullet					
No (ref)	43 (28.9)	106 (71.1)	Ref	Ref	—
Yes	75 (38.7)	119 (61.3)	1.55 (0.98–2.45)	1.11 (0.63–1.94)	0.724
Friends died by gunshot					
No (ref)	76 (31.5)	165 (68.5)	Ref	Ref	—
Yes	42 (41.2)	60 (58.8)	1.52 (0.94–2.45)	2.22 (1.13–4.38)	0.021*
Seen dead bodies					
No (ref)	57 (28.5)	143 (71.5)	Ref	Ref	—
Yes	61 (42.7)	82 (57.3)	1.87 (1.19–2.93)	1.55 (0.82–2.90)	0.174
Seen execution					
No (ref)	60 (30.3)	138 (69.7)	Ref	Ref	—
Yes	58 (40.0)	87 (60.0)	1.53 (0.98–2.40)	0.96 (0.52–1.77)	0.906
Fled residence					
No (ref)	23 (25.0)	69 (75.0)	Ref	Ref	—
Yes	95 (37.9)	156 (62.1)	1.83 (1.07–3.12)	1.24 (0.63–2.45)	0.528
Threatened by armed personnel					
No (ref)	53 (29.0)	130 (71.0)	Ref	Ref	—
Yes	65 (40.6)	95 (59.4)	1.68 (1.07–2.63)	1.20 (0.68–2.11)	0.524
Experienced transport accident					
No (ref)	54 (25.7)	156 (74.3)	Ref	Ref	—
Yes	64 (48.1)	69 (51.9)	2.68 (1.69–4.24)	2.30 (1.05–5.03)	0.037*
Witnessed severe transport accident					
No (ref)	52 (26.5)	144 (73.5)	Ref	Ref	—
Yes	66 (44.9)	81 (55.1)	2.26 (1.43–3.55)	1.13 (0.50–2.52)	0.773
Family died in transport accident					
No (ref)	90 (32.3)	189 (67.7)	Ref	Ref	—
Yes	28 (43.8)	36 (56.2)	1.63 (0.94–2.84)	0.98 (0.49–1.96)	0.964
Been robbed					
No (ref)	59 (29.2)	143 (70.8)	Ref	Ref	—
Yes	59 (41.8)	82 (58.2)	1.74 (1.11–2.74)	1.02 (0.57–1.84)	0.936
Non-family physical assault					
No(ref)	65 (31.4)	142 (68.6)	Ref	Ref	—
Yes	53 (39.0)	83 (61.0)	1.39 (0.89–2.19)	1.63 (0.87–3.03)	0.127
Family physical assault					
No (ref)	92 (32.6)	190 (67.4)	Ref	Ref	—
Yes	26 (42.6)	35 (57.4)	1.53 (0.87–2.70)	1.11 (0.54–2.25)	0.781
Home destruction due to violence					
No (ref)	90 (31.3)	198 (68.8)	Ref	Ref	—
Yes	28 (50.9)	27 (49.1)	2.28 (1.27–4.09)	1.83 (0.90–3.71)	0.094
Home invasion					
No (ref)	62 (30.9)	139 (69.1)	Ref	Ref	—
Yes	56 (39.4)	86 (60.6)	1.46 (0.93–2.29)	1.16 (0.65–2.06)	0.623

*p < 0.05

## Discussion

This study examined the factors associated with probable post-traumatic stress disorder (PTSD) among university students in Mogadishu, Somalia, and found that 34.4% of participants met the criteria for probable PTSD. This proportion is markedly higher than the global average of 3.9% [26] and is consistent with findings from Somalia and other conflict-affected settings. A study among internally displaced persons (IDPs) in Mogadishu reported a PTSD prevalence of 32%, while another investigation identified a prevalence of 29.9% among conflict-exposed Somali populations [22, 27]. Comparable figures have also been documented in Ethiopia, where between 36% and 41% of individuals affected by armed conflict or displacement were found to have PTSD [28, 29]. These findings underscore the significant psychological impact of prolonged insecurity, violence, and under-resourced mental health systems in Somalia and the wider Horn of Africa.

While these findings highlight the substantial psychological toll of conflict, the use of a self-reported screening instrument—the PTSD Checklist for DSM-5 (PCL-5)—should be considered when interpreting the results. Self-report tools like the PCL-5 can capture a broader range of trauma-related symptoms compared to structured clinical interviews, which apply more stringent diagnostic criteria [30]. This may lead to higher estimates of probable PTSD. Additionally, in Somali cultural contexts, psychological distress is often expressed through physical symptoms such as pain or fatigue, which may lead to underreporting or misclassification of PTSD symptoms [31, 32]. Therefore, both the measurement approach and cultural expression of distress should be considered when looking at the estimates of burden of PTSD in this population.

A critical finding in this study was the near-universal exposure to traumatic events among participants, with only four paticipants unexposed, and certain types of trauma demonstrating association with probable PTSD. Losing a friend to gun violence more than doubled the odds of probable PTSD, underscoring the profound psychological toll of sudden and violent bereavement. This aligns with prior research demonstrating that interpersonal loss, especially in violent circumstances, intensifies trauma responses and increases vulnerability to PTSD [33]. Similarly, experiencing a transportation accident was associated with significantly increased probable PTSD risk. This is consistent with studies linking transportation-related trauma to hypervigilance and persistent fear responses [34, 35]. Given that university students in Mogadishu rely heavily on public transport, their heightened exposure to road-related trauma may contribute to elevated PTSD risk.

This study found that female participants had five times higher odds of probable PTSD than males, corroborating global evidence that women are more susceptible to PTSD [36, 37]. In Somalia, this disparity is likely amplified by gender-specific stressors, including increased exposure to gender-based violence, domestic abuse, and societal stigma surrounding mental health [38, 39]. Women’s heightened vulnerability to PTSD may also stem from biological and psychological differences in stress response [40–42]. These findings emphasize the need for gender-sensitive mental health interventions tailored to the unique experiences of women in conflict-affected regions.

Despite the majority of participants reporting moderate to strong social support, no significant association was found between support levels and probable PTSD. This contrasts with earlier studies suggesting a protective effect of social connections [12, 13]. The limited variability in support levels may have reduced the ability to detect a meaningful association. Additionally, prolonged conflict in Somalia may have undermined the effectiveness of traditional support systems. Future research should explore how different forms of support—familial, peer, and institutional—interact with trauma exposure in similar settings [43].

## Conclusion

This study found that 34.4% of university students in Mogadishu met the criteria for probable PTSD. Significant factors associated with probable PTSD included the loss of a friend to gun violence, involvement in a transportation accident, and being female. These findings reflect not only individual trauma exposures but also the broader context of prolonged political instability and recurrent violence in Mogadishu, which create persistent insecurity and elevate the risk of trauma among youth.

Given the absence of university-based mental health services and the limited availability of care in the broader health system, these results underscore the urgent need to establish accessible, evidence-informed support for students. Universities should prioritize the development of basic psychosocial services, including grief and trauma counseling, while also advocating for strengthened referral pathways to external mental health providers. Gender-sensitive approaches are particularly important to address the higher vulnerability observed among female students. Moreover, targeted interventions for students affected by gun violence and transport-related trauma are needed. Given the limited availability of in-person services, online delivery of evidence-based interventions such as Eye Movement Desensitization and Reprocessing (EMDR) and trauma-focused Cognitive Behavioral Therapy (CBT) may provide feasible alternatives in conflict-affected Somali settings [44].

Further research should validate PTSD screening tools for the Somali context, explore gender-specific trauma experiences, and assess trauma exposure and PTSD symptoms among non-university youth. These efforts will support the development of locally relevant mental health strategies in resource-constrained, conflict-affected settings like Somalia.

### Study limitations

This study is subject to several limitations. Its cross-sectional design, reliance on self-reported data, and potential recall bias may affect the accuracy of reported trauma exposures. Although the PTSD Checklist for DSM-5 (PCL-5) is a widely used and validated screening tool, it is not a diagnostic instrument and does not independently assess the timing or duration of symptoms. Therefore, the findings represent cases of probable PTSD rather than clinically confirmed diagnoses, and there is a potential risk of misclassifying Acute Stress Disorder (ASD) as PTSD, particularly in the absence of clinical evaluation. Additionally, the applicability of the tool within the Somali context remains unverified, and the focus on university students limits the generalizability of the findings to the broader population.

## Data Availability

The datasets used and/or analyzed during the current study are available from the corresponding author on reasonable request.

## References

[1] APA. Diagnostic and Statistical Manual of Mental Disorders (5th ed). American Psychiatric Publishing. 2013: 970. Available from: https://cdn.website-editor.net/30f11123991548a0af708722d458e476/files/uploaded/DSM%2520V.pdf. Accessed 16 May 2021.

[2] Koenen KC, Ratanatharathorn A, Ng L, McLaughlin KA, Bromet EJ, Stein DJ et al. Posttraumatic stress disorder in the World Mental Health Surveys. Psychological Medicine. Cambridge University Press; 2017;47: 2260–74. Cited 6 Jun 2021. Available from:/pmc/articles/PMC6034513/. 10.1017/S0033291717000708 PMC6034513 28385165

[3] Ng LC, Stevenson A, Kalapurakkel SS, Hanlon C, Seedat S, Harerimana B et al. National and regional prevalence of posttraumatic stress disorder in sub-Saharan Africa: A systematic review and meta-analysis. PLoS Med. 2020;17(5):e1003090. Cited 31 Mar 2021. Available from: 10.1371/journal.pmed.1003090. 10.1371/journal.pmed.1003090 PMC7228043 32413027

[4] Farhood LF, Dimassi H. Prevalence and predictors for post-traumatic stress disorder, depression and general health in a population from six villages in South Lebanon. Soc Psychiatry Psychiatr Epidemiol. 2012 ;47(4):639–49. Cited 12 Apr 2023. Available from: https://pubmed.ncbi.nlm.nih.gov/21455787/. 10.1007/s00127-011-0368-6 21455787

[5] Pejuskovic B, Lecic-Tosevski D, Toskovic O. Longitudinal study of PTSD and depression in a war-exposed sample – comorbidity increases distress and suicide risk. Glob Psychiatry. 2020;3(1):64–71.

[6] Roberts B, Browne J. A systematic review of factors influencing the psychological health of conflict-affected populations in low- and middle-income countries. Glob Public Health. 2011;6:814–29. 20859816 10.1080/17441692.2010.511625

[7] McGinty G, Fox R, Ben-Ezra M, Cloitre M, Karatzias T, Shevlin M et al. Sex and age differences in ICD-11 PTSD and complex PTSD: An analysis of four general population samples. European Psychiatry. 2021 ;64(1). Cited 4 Mar 2023. Available from: /pmc/articles/PMC8581703/. 10.1192/j.eurpsy.2021.2239 PMC8581703 34602122

[8] Ayazi T, Lien L, Eide AH, Ruom MM, Hauff E. What are the risk factors for the comorbidity of posttraumatic stress disorder and depression in a war-affected population? A cross-sectional community study in South Sudan. BMC Psychiatry. 2012;12:175. 10.1186/1471-244X-12-175 PMC3534332 23083301

[9] Brunnet AE, Bolaséll LT, Weber JLA, Kristensen CH. Prevalence and factors associated with PTSD, anxiety and depression symptoms in Haitian migrants in southern Brazil. Int J Soc Psychiatry. 2018;64(1):17–25. Cited 4 Mar 2023. Available from: https://pubmed.ncbi.nlm.nih.gov/29082817/. 10.1177/0020764017737802 29082817

[10] Farhood LF, Fares S, Sabbagh R, Hamady C. PTSD and depression construct: Prevalence and predictors of co-occurrence in a south Lebanese civilian sample. Eur J Psychotraumatol . 2016;7(1):31509. Cited 26 Mar 2021. Available from: https://www.tandfonline.com/doi/full/.10.3402/ejpt.v7.31509 10.3402/ejpt.v7.31509 PMC4944596 27414815

[11] Nickerson A, Schick M, Schnyder U, Bryant RA, Morina N. Comorbidity of Posttraumatic Stress Disorder and Depression in Tortured, Treatment-Seeking Refugees. J Trauma Stress. 2017;30(4):409–15. Cited 26 Mar 2021. Available from: https://pubmed.ncbi.nlm.nih.gov/28763568/. 10.1002/jts.22205 28763568

[12] Dworkin ER, Ojalehto H, Bedard-Gilligan MA, Cadigan JM, Kaysen D. Social support predicts reductions in PTSD symptoms when substances are not used to cope: A longitudinal study of sexual assault survivors. J Affect Disord. 2018;229:135–40. 29310061 10.1016/j.jad.2017.12.042 PMC5807183

[13] Platt J, Keyes KM, Koenen KC. Size of the social network versus quality of social support: Which is more protective against PTSD? Soc Psychiatry Psychiatr Epidemiol. 2014;49(8):1279–86. Cited 2 Oct 2024 Available from: https://link.springer.com/article/10.1007/s00127-013-0798-4. 10.1007/s00127-013-0798-4 PMC7864543 24310782

[14] ACLED. ACLED Bringing clarity to crisis. Context Assessment: Heightened Political Violence in Somalia. 2023. Cited 4 Mar 2023. Available from: https://acleddata.com/2023/03/03/context-assessment-heightened-political-violence-in-somalia/.

[15] Im H, Swan LET, Warsame AH, Isse MM. Risk and protective factors for comorbidity of PTSD, depression, and anxiety among Somali refugees in Kenya. Int J Soc Psychiatry. 2020. 10.1177/0020764020978685 33300411

[16] Onyut LP, Neuner F, Ertl V, Schauer E, Odenwald M, Elbert T. Trauma, poverty and mental health among Somali and Rwandese refugees living in an African refugee settlement – an epidemiological study. Confl Health. 2009;3(1):1–16. 19470171 10.1186/1752-1505-3-6 PMC2695430

[17] Unfpa. Somali adolescent and youth . 2020. Available from: https://somalia.unfpa.org/sites/default/files/pub-pdf/Youth Report.pdf. Accessed 29 May 2022.

[18] Yousef L, Ebrahim O, AlNahr MH, Mohsen F, Ibrahim N, Sawaf B. War-related trauma and post-traumatic stress disorder prevalence among Syrian university students. Eur J Psychotraumatol. 2021 ;12(1). Cited 3 Apr 2023. Available from: https://pubmed.ncbi.nlm.nih.gov/34589173/. 10.1080/20008198.2021.1954774 PMC8475097 34589173

[19] Al-Shawi AF, Al-Hemiary NJ, Al-Diwan JK, Tahir DH. Post-Traumatic Stress Disorder among University Students in Baghdad: A Preliminary Report. Iraqi journal of community medicine . 2011;24(4). Cited 3 Apr 2023. Available from: https://www.iasj.net/iasj/article/61173.

[20] Nwoga C, Audu M, Obembe A. Prevalence and correlates of posttraumatic stress disorder among medical students in the University of Jos, Nigeria. Niger J Clin Pract. 2016;19(5):595–9. Cited 3 Apr 2023. Available from: https://pubmed.ncbi.nlm.nih.gov/27538546/. 10.4103/1119-3077.188704 27538546

[21] Charan J, Biswas T. How to Calculate Sample Size for Different Study Designs in Medical Research? Indian J Psychol Med. 2013;35(2):121. Cited 31 Oct 2024. Available from: https://pmc.ncbi.nlm.nih.gov/articles/PMC3775042/. 10.4103/0253-7176.116232 PMC3775042 24049221

[22] Ali M, Mutavi T, Mburu JM, Mathai M. Prevalence of Posttraumatic Stress Disorder and Depression Among Internally Displaced Persons in Mogadishu-Somalia. Neuropsychiatr Dis Treat. 2023;19:469. Cited 29 Sep 2024. Available from: /pmc/articles/PMC9985393/. 10.2147/NDT.S398423 PMC9985393 36879949

[23] Forkus SR, Raudales AM, Rafiuddin HS, Weiss NH, Messman BA, Contractor AA. The Posttraumatic Stress Disorder (PTSD) Checklist for DSM–5: A Systematic Review of Existing Psychometric Evidence., Clin, Psychol. (New York). 2022;30(1):110. Cited 27 Mar 2025. Available from: https://pmc.ncbi.nlm.nih.gov/articles/PMC10292741/. 10.1037/cps0000111 PMC10292741 37378352

[24] Ibrahim H, Ertl V, Catani C, Ismail AA, Neuner F. Trauma and perceived social rejection among Yazidi women and girls who survived enslavement and genocide. BMC Med. 2018;16(1):1–11. Cited 16 Jul 2025. Available from: https://bmcmedicine.biomedcentral.com/articles/10.1186/s12916-018-1140-5. 10.1186/s12916-018-1140-5 PMC6136186 30208905

[25] Caron J. [A validation of the Social Provisions Scale: the SPS-10 items]. Sante Ment Que. 2013;38(1):297–318. Cited 24 Jun 2023 Available from: https://pubmed.ncbi.nlm.nih.gov/24337002/. 10.7202/1019198ar PMC5031489 24337002

[26] Kessler RC, Aguilar-Gaxiola S, Alonso J, Benjet C, Bromet EJ, Cardoso G et al. Trauma and PTSD in the WHO World Mental Health Surveys. Eur J Psychotraumatol . 2017;8(sup5). Cited 3 Apr 2023. Available from: https://pubmed.ncbi.nlm.nih.gov/29075426/. 10.1080/20008198.2017.1353383 PMC5632781 29075426

[27] Salad AM, Malik SMMR, Ndithia JM, Noor Z, Madeo M, Ibrahim M. Prevalence of mental disorders and psychological trauma among conflict- affected population in somalia: a cross-sectional study. Front Public Health. 2023;11:1219992. 37829096 10.3389/fpubh.2023.1219992 PMC10565346

[28] Ali D, Azale T, Wondie M, Tadesse J. About Six in Ten Survivors of the November 2020 Maikadra Massacre Suffer from Posttraumatic Stress Disorder, Northwest Ethiopia. Psychol Res Behav Manag. 2022;15:251–60. Cited 29 Sep 2024. Available from: https://www.tandfonline.com/action/journalInformation?journalCode=dprb20. 10.2147/PRBM.S338823 PMC8845114 35177942

[29] Madoro D, Kerebih H, Habtamu Y, G/tsadik M, Mokona H, Molla A et al. Post-traumatic stress disorder and associated factors among internally displaced people in South Ethiopia: A cross-sectional study. Neuropsychiatr Dis Treat. 2020;16:2317–26. Cited 29 Sep 2024. Available from: https://www.tandfonline.com/action/journalInformation?journalCode=dndt20. 10.2147/NDT.S267307 PMC7548318 33116530

[30] McDonald SD, Calhoun PS. The diagnostic accuracy of the PTSD checklist: a critical review. Clin Psychol Rev. 2010;30(8):976–87. Cited 18 Nov 2024. Available from: https://pubmed.ncbi.nlm.nih.gov/20705376/. 10.1016/j.cpr.2010.06.012 20705376

[31] Bentley JA, Thoburn JW, Stewart DG, Boynton LD. The indirect effect of somatic complaints on report of posttraumatic psychological symptomatology among Somali refugees. J Trauma Stress. 2011;24(4):479–82. Cited 18 Nov 2024. Available from: https://pubmed.ncbi.nlm.nih.gov/21755542/. 10.1002/jts.20651 21755542

[32] Westermeyer JJ, Campbell R, Lien R, Spring M, Johnson DR, Butcher J et al. HADStress: A somatic symptom screen for posttraumatic stress among somali refugees. Psychiatric Services. 2010;61(11):1132–7. Cited 18 Nov 2024. Available from: 10.1176/ps.2010.61.11.1132. 10.1176/ps.2010.61.11.1132 21041353

[33] Affrunti NW, Suárez L, Simpson D. Community violence and posttraumatic stress disorder symptoms in urban youth: The moderating influence of friend and parent support. J Community Psychol. 2018;46(5):636–50. Cited 27 Nov 2024. Available from: https://onlinelibrary.wiley.com/doi/full/10.1002/jcop.21963. 10.1002/jcop.21963 31682288

[34] Birkeland MS, Skar AMS, Jensen TK. Do different traumatic events invoke different kinds of post-traumatic stress symptoms? Eur J Psychotraumatol. 2021;12(Suppl):1866399. Cited 27 Nov 2024. Available from: https://pmc.ncbi.nlm.nih.gov/articles/PMC8018401/.

[35] Jenkins EJ, Wang E, Turner L. Traumatic events involving friends and family members in a sample of African American early adolescents. Am J Orthopsychiatry. 2009;79(3):398–406. Cited 27 Nov 2024. Available from: https://pubmed.ncbi.nlm.nih.gov/19839677/. 10.1037/a0016659 19839677

[36] Andualem F, Melkam M, Takelle GM, Nakie G, Tinsae T, Fentahun S, et al. Prevalence of posttraumatic stress disorder and associated factors among displaced people in africa: a systematic review and meta-analysis. Front Psychiatry. 2024;15:1336665. 38516263 10.3389/fpsyt.2024.1336665 PMC10956696

[37] Tesfaye AH, Sendekie AK, Kabito GG, Engdaw GT, Argaw GS, Desye B et al. Post-traumatic stress disorder and associated factors among internally displaced persons in Africa: A systematic review and meta-analysis. PLoS One. 2024;19(4):e0300894. Cited 9 Dec 2024. Available from: https://journals.plos.org/plosone/article?id=10.1371/journal.pone.0300894. 10.1371/journal.pone.0300894 PMC10984478 38557637

[38] Bangura I. Trapped in violence and uncertainty: Patriarchy, Women, and the conflict in Somalia. Afr Confl Peacebuilding Rev. 2021;11(1):80–103.

[39] Dahie HA, Dakane MM, Hassan BS. Prevalence, patterns, and determinants of gender-based violence among women and girls in IDP camps, Mogadishu-Somalia. J Migr Health. 2023;8:100193. Cited 9 Dec 2024. Available from: https://pmc.ncbi.nlm.nih.gov/articles/PMC10450962/. 10.1016/j.jmh.2023.100193 PMC10450962 37637858

[40] Lehner M, Skórzewska A, Wisłowska-Stanek A. Sex-Related Predisposition to Post-Traumatic Stress Disorder Development-The Role of Neuropeptides. Int J Environ Res Public Health. 2021;19(1). Cited 1 Oct 2024. Available from: https://pubmed.ncbi.nlm.nih.gov/35010574/. 10.3390/ijerph19010314 PMC8750761 35010574

[41] Olff M. Sex and gender differences in post-traumatic stress disorder: an update. Eur J Psychotraumatol. 2017;8(sup4):4. Cited 1 Oct 2024. Available from: https://www.tandfonline.com/doi/abs/10.1080/20008198.2017.1351204.

[42] Ramikie TS, Ressler KJ. Mechanisms of Sex Differences in Fear and Posttraumatic Stress Disorder. Biol Psychiatry . 2018;83(10):876–85. Cited 1 Oct 2024. Available from: https://pubmed.ncbi.nlm.nih.gov/29331353/. 10.1016/j.biopsych.2017.11.016 29331353

[43] Alipour F, Ahmadi S. Social support and Posttraumatic Stress Disorder (PTSD) in earthquake survivors: a systematic review. Soc Work Ment Health. 2020;18(5):501–14. Cited 2 Oct 2024. Available from: https://www.tandfonline.com/doi/abs/10.1080/15332985.2020.1795045.

[44] Wang Y, Li X. Online Eye Movement Desensitization and Reprocessing for the Treatment of Post-Traumatic Stress Disorder. Alpha Psychiatry. 2024;25(1):113. Cited 27 Aug 2025. Available from: https://pmc.ncbi.nlm.nih.gov/articles/PMC11114197/. 10.5152/alphapsychiatry.2024.231411 PMC11114197 38799494

